# Composition and Function of T Cell Subpopulations Are Slow to Change Despite Effective Antiretroviral Treatment of HIV Disease

**DOI:** 10.1371/journal.pone.0085613

**Published:** 2014-01-21

**Authors:** Brinda Emu, Walter J. Moretto, Rebecca Hoh, Melissa Krone, Jeffrey N. Martin, Douglas F. Nixon, Steven G. Deeks, Joseph M. McCune

**Affiliations:** 1 Department of Medicine, Yale University, New Haven, Connecticut, United States of America; 2 Gladstone Institute of Virology and Immunology, San Francisco, California, United States of America; 3 Positive Health Program, University of California San Francisco, San Francisco, California, United States of America; 4 Department of Epidemiology and Biostatistics, University of California San Francisco, San Francisco, California, United States of America; 5 Division of Experimental Medicine, University of California San Francisco, San Francisco, California, United States of America; University Hospital Zurich, Switzerland

## Abstract

The ability to reconstitute a normal immune system with antiretroviral therapy in the setting of HIV infection remains uncertain. This study aimed to characterize quantitative and qualitative aspects of various T cell subpopulations that do not improve despite effective ART. CD4∶CD8 ratio was evaluated in HIV-infected subjects with viral loads >10,000 copies/µl (“non-controllers”, n = 42), those with undetectable viral loads on ART (“ART-suppressed”, n = 53), and HIV-uninfected subjects (n = 22). In addition, T cell phenotype and function were examined in 25 non-controllers, 18 ART-suppressed, and 7 HIV-uninfected subjects. CD4∶CD8 ratio in non-controllers, ART-suppressed, and HIV-uninfected subjects was 0.25, 0.48, and 1.95 respectively (P<0.0001 for all comparisons). The increased ratio in ART-suppressed compared to non-controllers was driven by an increase of CD4+ T cells, with no change in the expanded CD8+ T cell population. Expansion of differentiated (CD28−CD27−CD45RA+/−CCR7−) T cell subpopulations persisted despite ART and minimal changes were noted in naïve T cell frequencies over time. Increased number of CD8+CD28− T cells and increased CD8+ CMV-specific T cell responses were associated with a decreased CD4∶CD8 ratio. Measures of T cell function demonstrated persistence of high frequencies of CD8+ T cells producing IFN–γ. Lastly, though all CD8+ subpopulations demonstrated significantly lower Ki67 expression in ART-suppressed subjects, CD4+ T cell subpopulations did not consistently show this decrease, thus demonstrating different proliferative responses in the setting of T cell depletion. In summary, this study demonstrated that CD4∶CD8 ratios remained significantly decreased and naïve T cell numbers were slow to increase despite long-term viral suppression on ART. In addition, there is a evidence of differential regulation of the CD4+ and CD8+ T cell subpopulations, suggesting independent homeostatic regulation of the two compartments.

## Introduction

HIV infection directly impacts the immune system by depleting CD4+ T cells, thereby preventing the generation and maintenance of effective antigen-specific T and B cell responses against exogenous antigens. Uncontrolled viral replication results in not only decreases in CD4+ T cells but also increases in CD8+ T cells and, correspondingly, a lower CD4∶CD8 T cell ratio [1]. A decrease in the CD4∶CD8 ratio has been associated with increased mortality in the general population, particularly in the elderly [2], [3]. Uncontrolled HIV replication also causes a decrease in CD4+ and CD8+ naïve T cell numbers, and a concomitant increase in the proportion of highly differentiated effector T cells, particularly the CD28− T cell subpopulation [4]–[7]. Decreases in naïve T cells may be due to decreased thymic output and/or to the recruitment of naïve T cells into the memory/effector cell compartments through antigen-specific stimulation [8]–[10]. Decreases in naive T cells, particularly in CD28+ cells, have also been reported in the elderly and have been associated with increased mortality [11].

Effective antiretroviral therapy (ART) results in a complete or near-complete inhibition of HIV replication, sustained decreases in T cell activation, and slow but typically sustained increases in CD4+ T cell counts. These changes have led to the dramatically significant decreases in AIDS-related conditions and mortality [12]–[15]. Though the immunologic and clinical benefits of ART cannot be doubted, the degree to which ART can fully “normalize” immune function is less clear. In addition, there remains an increased incidence of non-AIDS events among HIV-infected individuals on ART and the etiology of these events have not been fully elucidated. We therefore performed a comprehensive analysis of effectively treated subjects to find that a number of immunologic parameters associated with altered phenotype and dysfunction in individuals with uncontrolled HIV replication are, in fact, only minimally changed with ART, despite long-term suppression of viral replication to undetectable levels. We report here that, despite effective ART, many adults have persistently low CD4∶CD8 ratios driven by expanded CD8+ T cells, limited increases in naïve CD8+ T cell numbers and frequency, and a shift in differentiation/maturation status of CD8+ and to less degree CD4+ T cells toward a more differentiated phenotype.

## Materials and Methods

### Ethics Statement

All participants provided written informed consent and this research was approved by the institutional review board of the University of California, San Francisco.

### Study Design

Blood was obtained from individuals enrolled in SCOPE, a prospective longitudinal observational cohort study based at the University of California, San Francisco. Cryopreserved peripheral blood mononuclear cells (PBMCs) were used for the analyses described below.

Participants met criteria for one of the following three groups (Table 1a): (1) healthy “HIV-uninfected” individuals; (2) “non-controllers,” defined as individuals with plasma HIV RNA levels >10,000 copies/mL on and off therapy; and (3) “ART-suppressed,” defined as ART-treated individuals with undetectable plasma HIV RNA levels. A total of 117 individuals (22 HIV-uninfected, 42 non-controllers, and 53 ART-suppressed) were studied with respect to T cell parameters (i.e., the fraction and number of circulating CD4+ and CD8+ T cells, and their ratios). PBMCs from 50 unique individuals (7 HIV-uninfected, 25 non-controllers, and 18 ART-suppressed) were studied by multiparameter flow cytometry (Table 1b).

**Table 1:** Baseline Characteristics of Subjects.

	HIV-uninfected (N = 22)	Non-Controllers (N = 42)	ART- Suppressed (N = 53)
Age (Yrs)	37 (27–60)	44 (33–62) **	50 (35–64) ** !!
Years infected	NA	18 (6–29)	18 (8–26)
CD4 count/µL	943 (582–1723)	249 (5–857)***	478 (127–899)*** !!!
CD4 (%)	48 (31–56)	13 (1–38) ***	22 (9–47)*** !!
CD8 count/µL	486 (252–1031)	973 (79–4584)***	1016 (272–3169)***
CD4∶CD8 Ratio	1.951(1.029–3.574)	0.247 (0.015–0.857)***	0.481 (0.151–1.652)*** !!!
Log viral load	NA	4.24(4.00–5.61)	1.88 !!!

** = p<0.002;

*** = p<0.0001 compared with HIV-uninfected group.

!! = p<0.0005;

!!! = p<0.0001 compared with non-controller group.

Medians (ranges) reported.

Within the ART-suppressed group, fifteen individuals remained durably viral suppressed on ART (no single VL>500 copies) for at least one additional year. PBMCs from both timepoints were analyzed using flow cytometry.

### Cytokine flow cytometry

**Figure 1:**
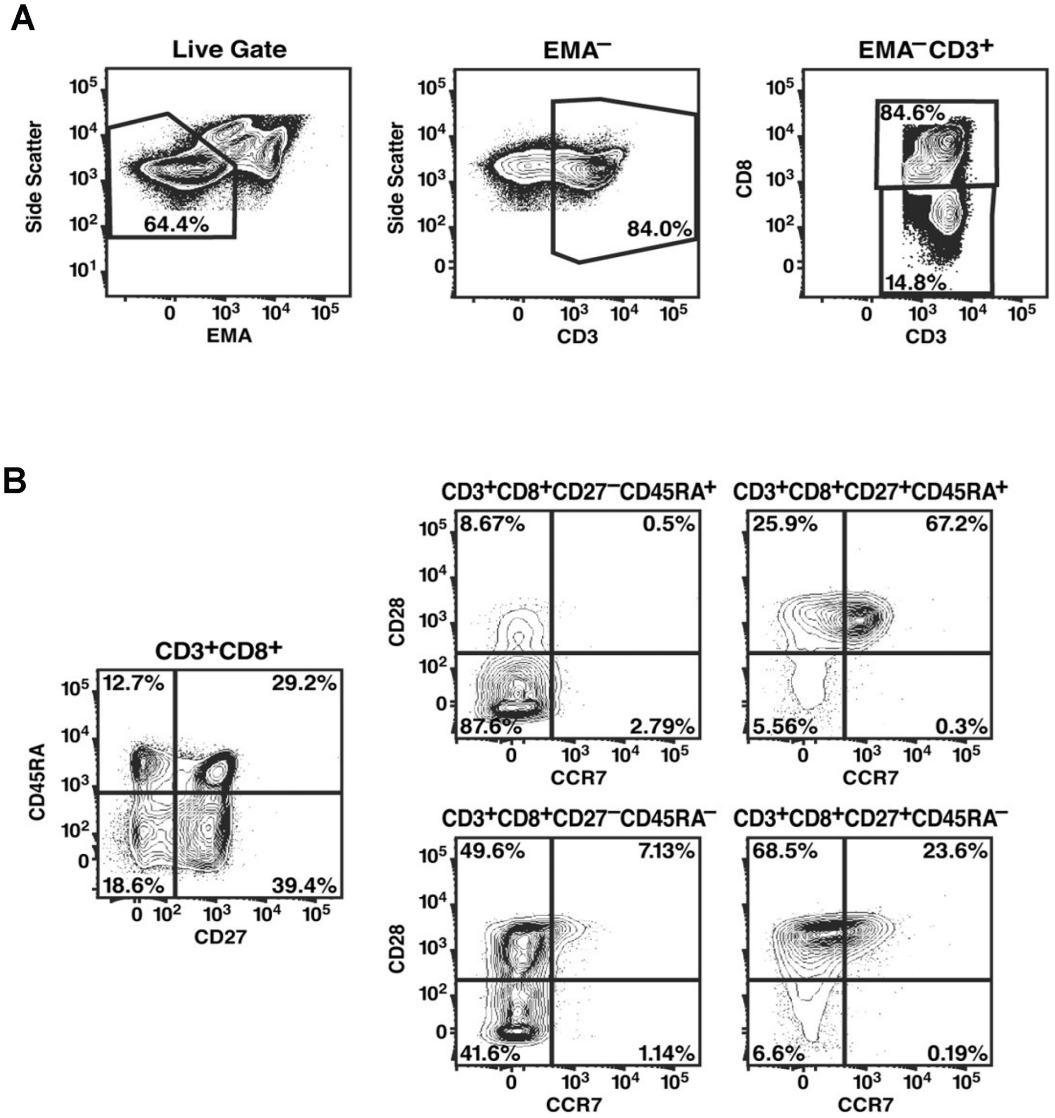
Gating of T cell populations. PBMCs stained and captured by FACSDIVA and analyzed FlowJo software. 1–2 million PBMCs were stimulated and stained, and all events captured for analysis. EMA staining was used to eliminate dead/dying cells from further analysis. EMA^−^ T cells were defined by side scatter and then by CD3 expression (A). EMA^−^ T cells that were CD3^+^CD8^−^ or CD3^+^CD8^+^ cells were designated as CD4^+^ and CD8^+^ T cells, respectively. The CD4^+^ and CD8^+^ T-cell populations were each divided into subpopulations based on their expression of CD45RA, CD27, CD28, and CCR7 (B). Gating utilized Fluorescence Minus One (FMO) strategy.

Cryopreserved PBMCs were re-suspended in RPMI 1640 medium with 15% fetal calf serum, rested overnight, then stimulated with 5 µg/mL staphylococcal enterotoxin B (SEB), 5 µg/mL overlapping Gag peptide pools (15-amino-acid peptides overlapping by 11 amino acids of HIV-1 p55 Gag; BD Biosciences, San Jose, CA) or 5 µg/mL overlapping pp65 peptide pools (15-amino-acid peptides overlapping by 11 amino acids of CMV pp65; BD Biosciences, San Jose, C A), or left un-stimulated, and incubated for 16 h at 37°C with 5 µg/ml brefeldin A one hour after the beginning of incubation. Cells were washed in PBS containing 2 mM EDTA, and then with PBS containing 1% bovine serum albumin (Sigma-Aldrich), and stained with a fluorescently labeled antibodies against the following cell surface markers: anti-CD45RA-PE (BD Biosciences), anti-CCR7-PE-Cy7 (BD Biosciences), anti-CD28-APC (BD Biosciences), anti-CD27-APC-Cy7 (eBiosciences, San Diego, CA), and anti-CD8-Cascade Blue (Dr. Nicole Blumgarth, University of California, Davis). For discrimination of dead cells, 5 µg/ml ethidium monoazide (EMA; Molecular Probes, Eugene, OR) was included in the cocktail of antibodies [16], [17]. Cells were exposed to a 40 W fluorescent light bulb for 5 min prior to being washed with PBS containing 1% bovine serum albumin, fixed in 1% paraformaldehyde, and permeabilized in FACS permeabilizing solution (BD Biosciences). Cells were then incubated with anti-CD3-PE-Texas Red (Beckman Coulter, Fullerton, CA) and either anti-IFN-γ-FITC, anti-IL-2-FITC, or anti-Ki67-FITC (BD Biosciences). Data were collected on a FACSDiVa (BD Biosciences) flow cytometer and analyzed using FlowJo software (Tree Star, San Carlos, CA) (Fig. 1).

Fifteen of the ART-suppressed individuals remained on ARV with sustained viral suppression. Longitudinal samples of cryopreserved PBMCs were obtained from these individuals and flow cytometric analysis was performed on samples from both time points. PBMCs were thawed, processed, and stimulated as described above, with the Live/Dead Aqua Fixable Dead Cell Stain Kit (Invitrogen). The following fluorescently labeled antibodies were used: anti-CD45RA-ECD, anti-CCR7-APC, anti-CD28-PE, anti-CD27-APC-Cy7, anti-CD8-PECy5.5, anti-CD4 Pacific Blue, anti-CD3-Alexa 700 and either anti-IFN-PE-Cy7, anti-IL-2-FITC, or anti-Ki67-FITC. Data were collected on a LSRII (BD Biosciences) and analyzed using FlowJo.

### Statistical Analysis

Differences in variables between any two patient groups were analyzed using the Mann-Whitney U test. Spearman's rank correlation was used to determine correlations between variables. For longitudinal time points within the ART-suppressed group, the Student paired t-test was used to evaluate changes over time.

## Results

### The CD4∶CD8 T cell ratio was partially restored with ART with persistent CD8+ T cell expansion

CD4+ and CD8+ T cell counts were evaluated in 22 HIV-uninfected adults, 42 non-controllers (HIV RNA levels>10,000 copies), and 53 ART-suppressed individuals (Table 1). Individuals in both non-controller and ART-suppressed groups had been infected with HIV for a median of 18 years. The median log plasma HIV RNA levels in the non-controllers was 4.24, and median CD4+ T cell counts in the non-controllers and ART-suppressed groups were 249 and 478 cells/mm^3^, respectively (p<0.0001). There was no significant difference in CD8+ T cell counts between non-controllers and ART-suppressed groups.

**Figure 2:**
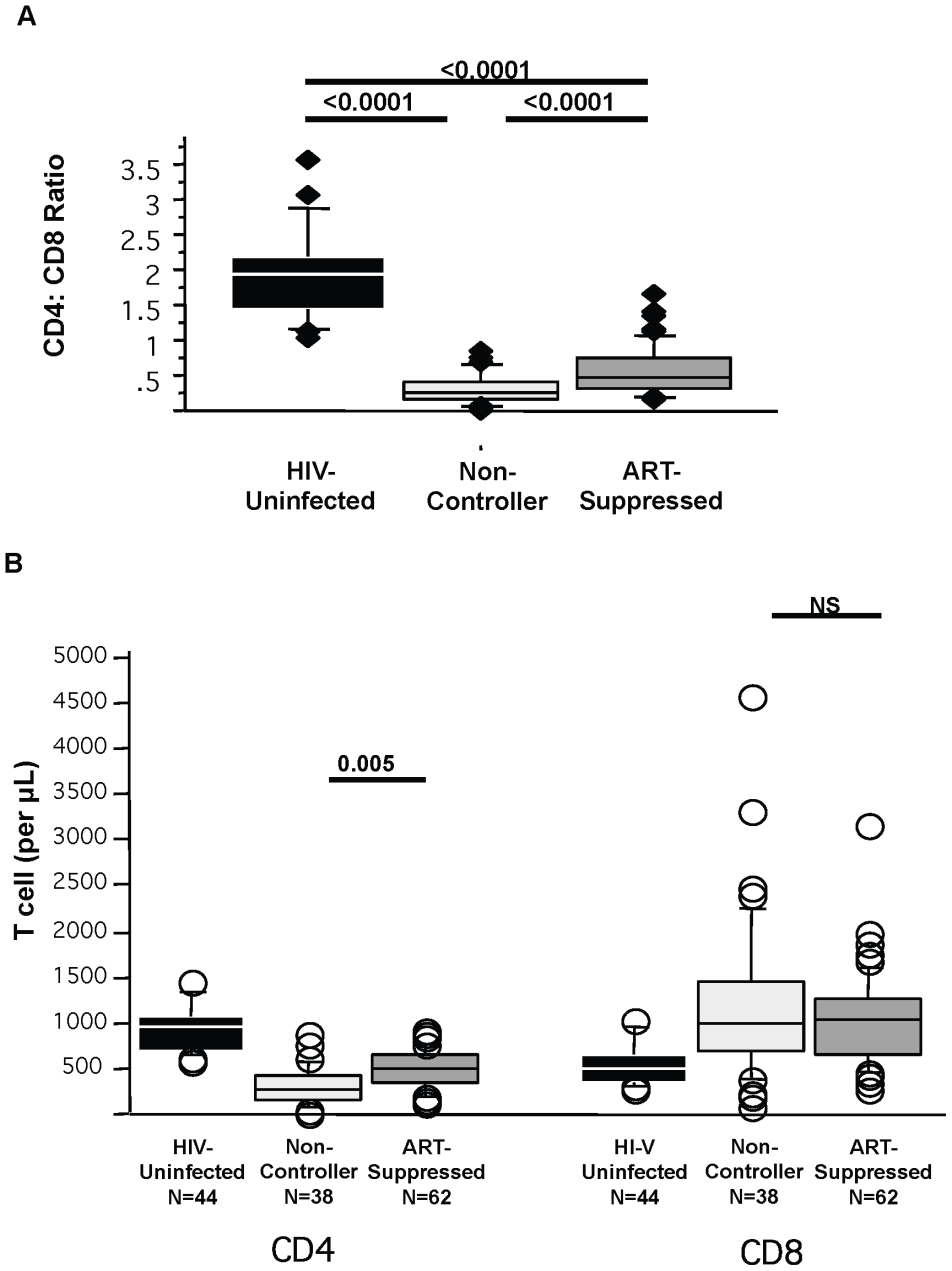
CD4∶CD8 T cell ratio. CD4+ and CD8+ T cell counts were enumerated among different patient groups. Non-controllers are HIV infected individuals with ongoing viral replication. ART-suppressed are HIV-infected individuals with undetectable peripheral viral loads on antiretroviral therapy. HIV-uninfected individuals are controls. CD4∶CD8 T cell ratios (A) and individual CD4 and CD8 T cell counts (B) are shown.

In HIV-uninfected individuals, the median CD4∶CD8 ratio of 1.95 (Fig. 2a) was as expected in healthy individuals [18], [19]. Consistent with previous reports, untreated HIV-infected non-controllers showed a markedly lower CD4∶CD8 ratio of 0.25 (p<0.0001). This lower ratio was due to both significantly lower CD4+ T cell counts and significantly higher CD8+ T cell counts (Fig. 2b). ART-suppressed individuals had a median CD4∶CD8 ratio of 0.481 (p<0.0001 compared to non-controllers and to HIV-uninfected). The higher ratio in the ART-suppressed group compared to non-controllers was due entirely to higher CD4+ T cells counts (p = 0.005) with no difference in CD8+ T cell counts (p = 0.82). Thus, despite increase in the CD4+ T cell count and in CD4∶CD8 ratio with antiretroviral treatment, complete normalization of the ratio did not occur due to a persistently expanded population of CD8+ T cells. There was no relationship between the CD4∶CD8 T cell ratio and the duration of suppression on ART, self-reported duration of HIV infection, or CD4 nadir (unpublished data).

### Demographics of individuals undergoing multiparameter flow cytometric analysis

A separate cohort of 7 HIV-uninfected, 25 non-controllers, and 18 ART-suppressed individuals was studied by flow cytometry (Table 2). Both groups were of similar age and had been HIV-infected for a comparable period of time. Median log plasma HIV RNA levels was 4.47 among the non-controllers. Median duration of ART therapy was 3.5 years (range 1.0 to 6.9 years) in the ART-suppressed group. In the non-controllers and ART-suppressed groups, the median CD4+ T counts were 270 and 487 cells/mm^3^ respectively (p<0.0001). Again, there was no significant difference in CD8+ T cell counts between non-controllers and ART-suppressed groups. Seven HIV-uninfected individuals were also evaluated, but baseline demographic data, CD4+ and CD8+ T cell numbers were not available for this group. Of note, ART-suppressed individuals, prior to initiation of ART, had CD4+ T cell count of 230, CD8+ T cell count of 655, and CD4∶CD8 ratio of 0.22. Thus, individuals suppressed on ART had comparable baseline T cell parameters to non-controllers.

**Table 2:** Baseline Characteristics of Subjects Studied by Flow Cytometry.

	Non-Controllers (N = 25)	ART-Suppressed (N = 18)
Age (Yrs)	44 (33–66)	47 (32–61)
Years infected	15 (3–25)	10 (6–20)
CD4 count/µL	270 (10–687)	482 (300–1079)***
CD4 percentage	12 (3–32)	21 (9–47) ***
CD8 count/µL	873 (230–2644)	1023(453–1693)
CD4∶CD8 ratio	0.20 (0.03–0.86)	0.44 (0.21–1.52)**
Log viral load	4.47 (4.02–>5.70)	1.88 ***
Years on suppressive ART	NA	3.5 (1.0–6.9)
*Pre-ART VL*	*NA*	*4.74 (3.88–>5.70)*
*Pre-ART CD4*	*NA*	*230 (26–601)*
*Pre-ART CD8*	*NA*	*655 (433–1653)*
*Pre-ART CD4∶CD8*	*NA*	*0.22 (0.07–0.58)*

** = p<0.007;

*** = p<0.0001 between ART-suppressed and non-controllers groups.

### Suppression of viremia does not result in differences in composition of CD8+ T cell subpopulations

**Figure 3:**
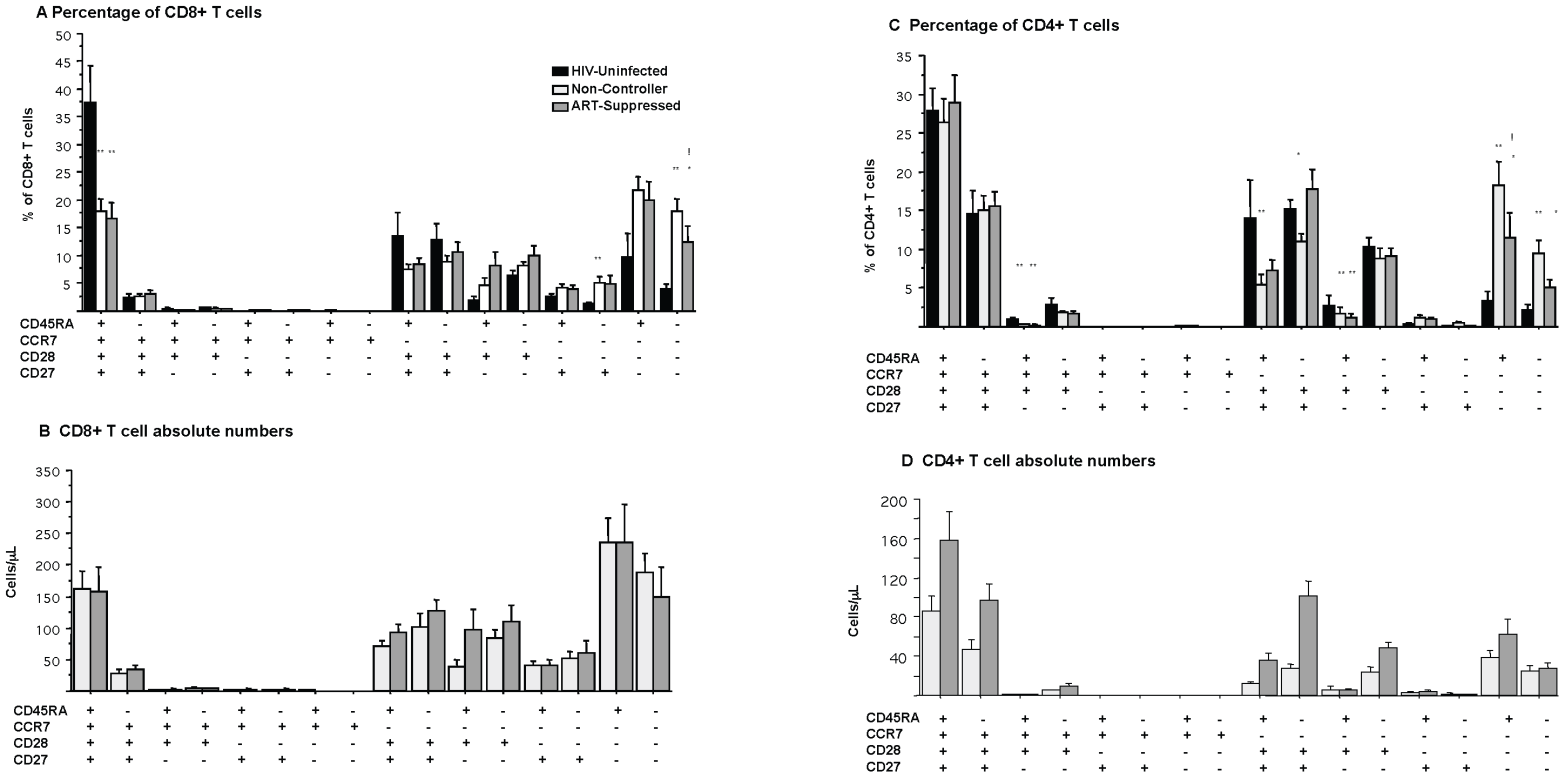
Subpopulations of CD4+ and CD8+ T cells. CD4+ and CD8+ T cell subpopulations were characterized with surface staining for CD27, CD28, CCR7, and CD45RA. Each of the sixteen subpopulations is shown as a percentage of total CD8+ (A) or CD4+ (C) T cell populations. Also shown are the absolute numbers of CD8+ (B) and CD4+ (D) T cell subpopulations. * represents p<0.05 compared to HIV-uninfected population; ** p<0.01 compared to HIV-uninfected population; ! Is comparison between non-controllers and ART-suppressed.

It has previously been reported that HIV infection results in the loss of circulating naïve cell subpopulations [5], [17]. Using four markers of T cell maturation (CD27, CD28, CD45RA, and CCR7), sixteen unique subpopulations of CD4+ and CD8+ T cells subpopulations were evaluated amongst the three groups (Fig. 3, with naïve subpopulations progressing to highly differentiated subpopulations from left to right). The expression of four markers was used to define naïve T cell phenotypes, providing confidence that these cells represent true naive T cell populations [20]–[22]. CD8+ T cells that are CD27−CD28−CD45RA+CCR7− and CD27−CD28−CD45RA−CCR7− represent the most differentiated effector T cells, capable of secreting high levels of interferon-γ (IFN-γ) and small amounts of IL-2 [20]–[22]. Subpopulations 3–8 among CD8+ T cells (subpopulations delineated as CD28−CCR7+ or CD27−CD28+CCR7+) and subpopulations 5–8 among CD4+ T cells (subpopulations delineated as CD28−CCR7+) (Fig. 3) were rarely present, either in HIV-uninfected or HIV-infected individuals, suggesting these phenotypes of T cells do not exist in peripheral circulation in appreciable numbers.

Upon evaluation of PBMCs from the two HIV-infected groups, the percentage of the most differentiated CD8+ T cell subpopulation (CD27−CD28−CD45RA−CCR7−) was found to be lower in the ART-suppressed than in non-controllers (p = 0.05, Fig. 3a). Strikingly, however, naïve CD8+ T cells (CD27+CD28+CD45RA+CCR7+) and most of the other circulating memory T cell subpopulations showed comparable frequencies in both the ART-suppressed and non-controller groups. This observation suggests that successful ART has limited effects on the relative distribution of CD8+ T cell subpopulations. Because there was no difference in total CD8+ T cell counts between the two groups, there also was no significant difference in the absolute number of naïve or highly differentiated CD8+ T cells (Fig. 3b).

In contrast to both HIV-infected groups, naïve CD8+ T cells made up the largest subpopulation of CD8+ T cells among healthy HIV-uninfected individuals, significantly higher than that seen in both HIV-infected groups. HIV-uninfected individuals also exhibited a lower proportion of most differentiated CD8+ T cell subpopulations and particularly of the most differentiated CD8+ T cell subpopulation (CD27−CD28−CD45RA−CCR7−).

### Suppression of viremia is associated with in higher numbers of circulating CD4+ T cell subpopulations

No difference between the frequency of naïve CD4+ T cells (CD27+CD28+CD45RA+CCR7+) was observed between non-controllers and ART-suppressed individuals (Fig. 3c). There was, however, a trend towards lower frequencies of the two most differentiated CD4+ T cell subpopulations among ART-suppressed individuals compared to non-controllers (p = 0.05 for CD27−CD28−CD45RA+CCR7−; p = 0.13 for CD27−CD28−CD45RA−CCR7−).

As expected, total number of circulating CD4+ T cells was higher in ART-suppressed individuals than non-controllers. Thus, despite the similarity in the *distribution* of subpopulations, ART-suppressed individuals had higher absolute numbers of most CD4+ T cell subpopulations compared to non-controllers (Fig. 3d), suggesting some recovery of both naïve and memory T cell populations. Of note, the subpopulation of CD4+ T cells with the largest increase was CD28+, suggesting a preferential increase in the production of naïve and central memory CD4+ T cells.

In comparison to HIV-uninfected individuals, there was no difference in the frequency of naïve CD4+ T cells (CD27+CD28+CD45RA+CCR7+) with either HIV-infected group. Similar to CD8+ T cell counts, the frequency of the more differentiated populations (CD27−CD28−CD45RA+CCR7− and CD27−CD28−CD45RA−CCR7−) was higher in both HIV-infected groups compared to HIV-uninfected individuals (p<0.05 for both subpopulations between HIV-uninfected and either HIV-infected group, Fig. 3c)

### Cytokine secretion among T cell subpopulations remains unchanged despite ART

**Figure 4:**
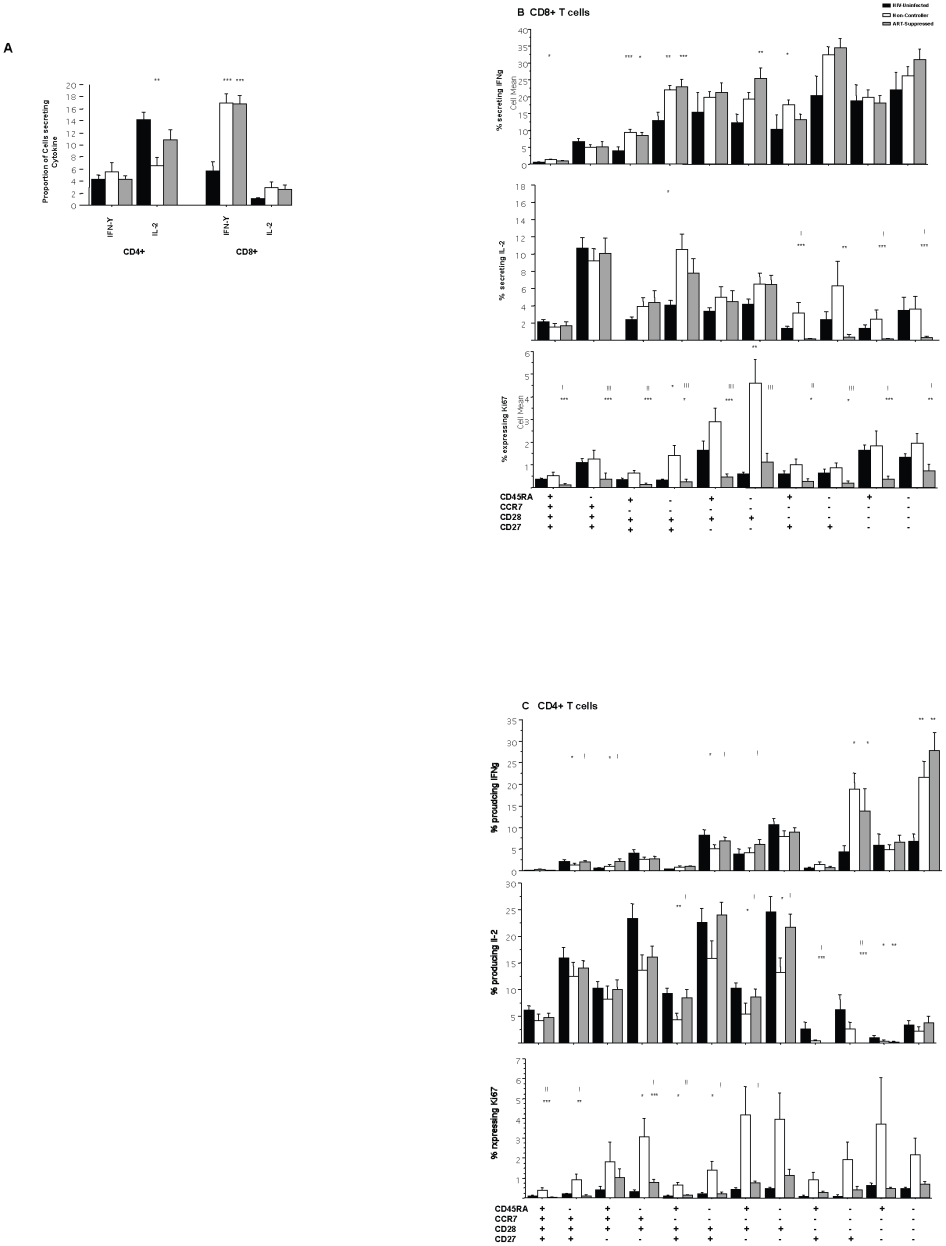
Function of CD4+ and CD8+ T cell populations. (A) Cytokine secretion in response to SEB. Cryopreserved PBMCs were thawed, rested, and stimulated with SEB or, alternatively, left unstimulated. The cells were then stained and analyzed on a FACS Diva. CD4+ and CD8+ cells secreting IFN-γ or IL-2 were enumerated by intracellular flow cytometry. (A)Cytokine secreting cells were quantified as [(cells secreting cytokine in response to SEB) – (unstimulated cells secreting cytokine)]. Results for both CD4+ and CD8+ T cells are shown. Compared with HIV-uninfected: **p<0.001, ***p<0.0005. (B,C) Cytokine secretion in response to SEB among T cell subpopulations and Ki67 expression in unstimulated T cells. CD8+ and CD4+ T cell subsets were stained simultaneously with CD27, CD28, CCR7, and CD45RA with representation of the twelve subpopulations of T cells that are present in circulation. Percentage of subpopulations producing cytokine (IFN-γ or IL-2) in response to SEB stimulation is shown. Percentage of cells with Ki67 expression is also depicted. Expression of Ki67 was analyzed in unstimulated cells only. Significance values: Compared with HIV-uninfected: *p<0.05, **p<0.01, ***p<0.005. Comparison between non-controllers and ART-suppressed: ! p<0.05, !! *p<0.01, !!! p<0.005.

T cell function was assessed, by measurement of IFN-γ and IL-2 production upon stimulation with Staphylococcal enterotoxin B (SEB). Level of CD8+ IFN-γ production in response to SEB stimulation among the ART-suppressed group was comparable to the non-controller group (p = 0.88). Both HIV-infected groups had significantly higher IFN-γ production compared with the HIV-uninfected population (p<0.005) (Fig. 4a). No significant difference in IL-2 production among CD8+ T cells was observed between the three groups. With regard to CD4+ T cells, non-controllers demonstrated significantly decreased IL-2 production among CD4+ T cells compared to HIV-uninfected individuals (p = 0.008). ART-suppressed individuals demonstrated a trend towards increased IL-2 production compared to non-controllers (p = 0.06) (Fig. 4a).

To determine whether particular T cell subpopulations were responsible for changes in T cell cytokine secretion, we evaluated the ability of each subpopulation to produce cytokines in response to SEB. The function of the ten CD8+ T cell subpopulations and twelve CD4+ T cell subpopulations that are present in the peripheral circulation (Fig. 3b, 3c) are shown (Fig. 4b, 4c). Among CD8+ T cells, non-controller and ART-suppressed groups demonstrated comparable IFN-γ production among *all* subpopulations. However, the ART-suppressed group showed a dramatic decrease in IL-2 production only in the CD8+CD28− T cell subpopulations compared to non-controllers. Interestingly, among CD4+ T cells, individuals with a viral load suppressed by ART demonstrated decreased IL-2 production in the CD28− subpopulations and *increased* IL-2 production in the CD4+ central memory subpopulations (i.e., CD28+CCR7−) compared to non-controllers.

We also measured HIV-specific and CMV-specific CD4+ and CD8+ T cell responses. There was no difference in IFN-γ or IL-2 production among CD4+ or CD8+ Gag-specific responses between the two HIV-infected subject groups. ART-suppressed individuals had increased CMV-specific CD4+ IFN-γ producing cells compared with non-controllers; all other responses were comparable. Of note, higher CMV-specific T cell responses were associated with a lower CD4∶CD8 ratio among ART-suppressed group.

Ki67, a marker of T cell activation and proliferation, was significantly decreased among CD4+ and CD8+ T cell subsets in the ART-suppressed group compared to non-controllers, as has been demonstrated by other groups [23]–[25]. We additionally show here that Ki67 expression among T cells is lower in every CD8+ T cell subpopulation in the ART-suppressed group compared to non-controllers. Ki67 was not significantly decreased in *all* CD4+ T cell populations in ART-suppressed group, suggesting continued activation and proliferation among CD4+ T cells that is not demonstrated in CD8+ T cell subpopulations.

When compared to the HIV-uninfected control population, the two HIV-infected groups demonstrated significant increases in IFN-γ secretion in several CD4+ and CD8+ T cell subpopulations. In addition, the ART-suppressed group exhibited a decrease in IL-2 secretion in the CD28− subpopulations and significant decrease in Ki67 expression in *all* CD8+ subpopulations with the exception of the CD45RA+CCR7−CD28+CD27− subpopulation (Fig. 4b) compared to HIV-uninfected individuals. In contrast to CD8+ T cells, Ki67 expression in CD4+ T cell subpopulations of ART-suppressed was comparable and, in some instances, higher than that seen in HIV-uninfected individuals for several CD4+ subpopulations (Fig. 4c). These differential responses in Ki67 expression and IL-2 secretion between the CD4+ and CD8+ T cell populations suggest different factors that impact homeostatic proliferation.

### Longitudinal evaluation of ART-suppressed individuals reveals improvement of the CD4∶CD8 ratio in some individuals

**Figure 5:**
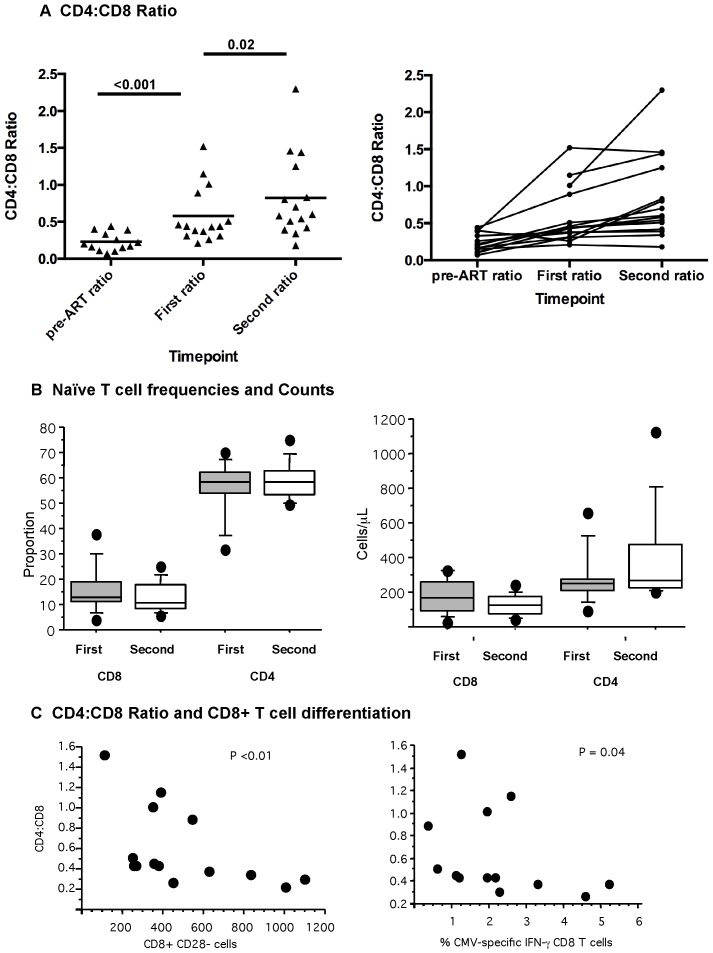
Longitudinal assessment of ART-suppressed subjects. (A) CD4∶CD8 ratio in ART-suppressed Individuals at three timepoints (pre-ART, first timepoint on ART and second timepoint on ART). Subjects remained on antiretroviral therapy with suppressed viral load between “first” and “second” timepoint on ART. (B) Naïve subpopulations defined as CD27+CD28hiCCR7+CD45RA+. Proportion and absolute number/ml are depicted for the first and second timepoint. (C) CD4∶CD8 ratio in those suppressed on ART is shown in relationship to proportion of CD8+CD28− cells and also in relationship to the proportion of CMV-specific CD8+ T cells.

Longitudinal evaluation was performed with PBMCs from 15 individuals who had remained on ART for a median of 3.5 *additional* years with documented viral loads below the limit of detection (Table 3). CD4, CD8 counts, CD4∶CD8 ratio and viral load measurements were also available at the pre-ART timepoint for these individuals. Initiation of ART resulted in a significant increase in CD4+ T cell counts (p<0.0001). Between the two time points on ART, total CD4+ T cell counts increased further (p = 0.02). Of note, no significant changes were noted in CD8+ T cell counts from pre-ART timepoint to the two additional measurements on ART. CD4∶CD8 ratio increased significantly from pre-ART timepoint to the first measurement on ART (p<0.0001) and again between the two timepoints on ART (p = 0.02), driven by CD4+ T cell count increases. However, when individual subjects were evaluated, overall gains in CD4∶CD8 ratio was driven by only a few individuals with substantial gains (Fig. 5a). The CD4∶CD8 ratio at the second timepoint on ART was associated with duration of HIV infection and time suppressed with ART (p = 0.06 for each variable) in a univariate analysis. However, when age and pre-ART CD4+ T cell count were included as covariates, the association was no longer significant; rather, the continued increase of CD4∶CD8 ratio with increased duration on ART was driven by a small number of individuals.

**Table 3:** Characteristics of ART-suppressed group over time.

	Pre-ART (N = 15)	First timepoint (N = 15)	Second timepoint (N = 15)
Age (Yrs)	47 (25–60)	50 (32–61)	56 (34–64)
Years infected	6 (1–16)	10 (6–20)	13 (7–23)
CD4 count/µL	226 (36–573)	482 (300–1079) **	462 (363 −1507) **, !
CD8 count/µL	655 (476–1653)!	1023 (484–1662)	828 (353–2192)
CD4∶CD8 ratio	0.20 (0.07–0.44)!	0.44 (0.21–1.52) **	0.60(0.18–2.30) **, !
Log viral load	4.74 (3.88–>5.70)	1.88 **	1.88 **
Yrs on suppressive ART	N/A	3.7(1.0–6.9)	5.5 (3.7–9.5)

For three subjects, CD8+ T cell counts were not available at the pre-ART timepoint. Thus, CD8+ T cell count and CD4∶CD8 ratio at the pre-ART timepoint is based on 12 subjects.

** = p<0.001 compared with pre-ART timepoint.

! = p = 0.02 when compared with first on-ART timepoint.

Of note, two markers of increased CD8+ T cell differentiation were associated with the CD4∶CD8 ratio: both expanded CD28− T cell numbers and increased CD8+ CMV-specific T cells were inversely associated with CD4∶CD8 ratio (p<0.01 and p = 0.04 respectively, Figure 5c).

There was no change in naïve T cell (CD27+CD28^hi^CD45RA+CCR7+) frequencies in either the CD4+ or CD8+ T cell subpopulations over time on ART (Fig. 5b). There was an increase in absolute number of naïve CD4+ T cells (p = 0.05), suggesting that recovery of naïve CD4+ T cell number can occur with increased time on ARV for a subset of individuals. Naïve CD8+ T cell recovery, on the other hand, is much less likely to occur, even with prolonged time on therapy.

## Discussion

In the vast majority of chronically HIV-infected subjects, immunologic consequences of uncontrolled HIV replication are dramatic, with loss of peripheral CD4+ T cells, reversal of CD4∶CD8 T cell ratio, and skewing of T cell subpopulations to more differentiated phenotypes. Each of these findings has been associated with rapid HIV disease progression and development of AIDS. [26]. Upon administration of effective ART, peripheral CD4+ T cell counts increase and there is a significant decrease in AIDS-related clinical conditions, but alterations in the distribution and function of certain T cell subpopulations persist. In this manuscript, we confirm findings of larger studies that there is a significant reduction of CD4∶CD8 T cell ratio in untreated HIV infection with a decrease in naïve CD4 and CD8 T cells. We additionally show that, in a cohort of effectively treated adults who received therapy for a median of 3.5 years, some aspects of immune phenotype remain unchanged: CD4∶CD8 ratios remain decreased as do naïve CD8+ T cell frequency, and there are persistent increases in the circulating number of CD8+ T cells with differentiated effector (CD28−) phenotypes. With increased time on ART, it is notable that a subset of individuals experienced appreciable gains in their CD4+ T cell counts and normalization of their CD4∶CD8 ratio; however, the majority did not show continued improvement in this latter parameter. In addition, we demonstrate that the CD4+ and CD8+ T cell populations demonstrate differential proliferative status within their respective subpopulations. We suggest here that the continued, persistent expansion of highly differentiated CD8+ T cells limits the ability to regain total CD4+ T cells, and naïve CD4+ and CD8+ T cells, and the CD4∶CD8 ratio.

The inability to normalize CD4∶CD8 ratios, regain naïve CD8+ T cells and the persistent expansion of highly differentiated CD8+ T cells despite the administration of effective ART may be due to one or more contributing forces, including: (1) impairment in production of naïve T cells; (2) ongoing antigenic stimulation/activation, with continued recruitment into the effector/memory T cell compartment; and (3) altered homeostatic mechanisms. In all these scenarios, substantive and irreversible damage to stromal microenvironments necessary for the maintenance of normal T cell homeostasis may be playing a role.

With regard to antigenic stimulation, it is likely that low level HIV production persists in tissue reservoirs, even when viral loads are suppressed to undetectable levels in peripheral blood [27]–[29]. Given the strong pro-inflammatory effects that HIV has on the immune system and observations that measurement of HIV persistence correlate with T cell activation, it is possible that ongoing HIV replication contributes to continued, albeit lower, levels of antigenic stimulation [30], [31]. In addition, the presence of other pathogens, including cytomegalovirus and gut microbial products, may be an important contributor to chronic T cell activation [32]–[36]. We have shown that CMV-specific CD4+ and CD8+ responses remain high in ART-suppressed individuals, and that higher CMV-specific responses are associated with a lower CD4∶CD8 ratio. This is consistent with findings by Appay et al showing that higher CMV-specific responses are associated with poor total and naive T cell recovery in ART-treated subjects [37], suggesting that existing highly differentiated CD8+ T cells directed against CMV, and potentially other antigens, contribute to poor T cell reconstitution.

The role of differential CD4+ and CD8+ T cell turnover was evaluated in this study by measuring proliferative capacity (i.e., Ki67 expression) in naive, memory, and effector T cell subpopulations. In comparison to HIV-uninfected subjects, Ki67 expression is substantially lower in CD8+ T cell subpopulations, but comparable to higher among CD4+ T cell populations, from ART-suppressed subjects. This suggests independent and differential homeostatic regulation mechanisms of the two T cell compartments. This is consistent with the observation that Ki67 levels are very low in the persistently expanded CD8+ T cell population, but slightly higher than normal in the depleted CD4+ T cell compartment. This observation is supported by studies that demonstrate that CD4+ T cell proliferation is driven by both CD4+ T cell depletion and viral load, and whereas CD8+ T cell proliferation is largely driven by viral load alone [38].

**Figure 6:**
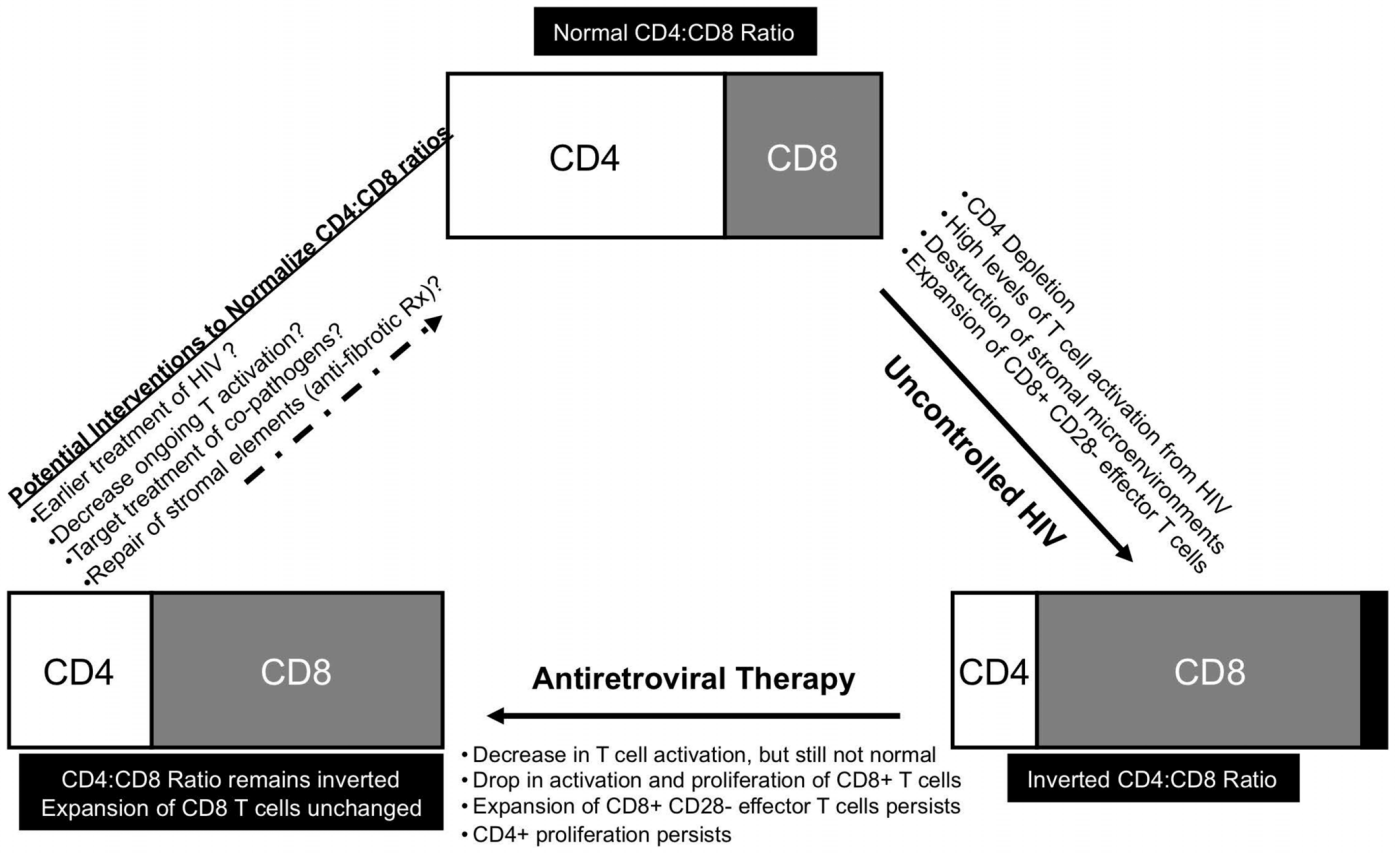
Schema of CD4∶CD8 ratio in HIV infection. Uncontrolled HIV infection results in depletion of CD4+ T cells with concomitant increase in CD8+ T cells, mostly of an effector phenotype. This process is largely driven by high levels of T cell activation. With introduction of antiretroviral therapy, there is a decrease in T cell activation and a resulting drop in activation and proliferation of CD8+ T cells. There is also some recovery of CD4+ T cells. However, an inverted CD4∶CD8 ratio persists due to persistently expanded CD8+ T cell population.

This study highlighted that despite long-term follow-up of HIV-infected subjects on ART, there is limited full recovery of naive T cell numbers, particularly naïve CD8+ T cells and limited recovery of the CD4∶CD8 ratio. A similar observation *has been reported* in the setting of patients with cancer who undergo ablation of T cells with high-dose chemotherapy followed by autologous hematopoietic stem cell transplantation (SCT) [39]. In this other clinical situation of profound T cell depletion, CD8+CD28− effector T cells rebound most quickly after SCT and to levels higher than prior to chemotherapy. However, even after naïve T cells begin to emerge, the expanded CD8+CD28− population remains the predominant subpopulation of T cells. This analogous clinical situation of profound T cell depletion and CD8+ effector T cell expansion also results in persistently lower CD4∶CD8 ratios and the inability to restore naïve T cells to normal numbers. We similarly show the persistence of highly differentiated T cells with a CD28−CD27−CD45RA+/−CCR7− phenotype with increased IFN-γ and decreased IL-2 production, which contribute to a large population of anergic, apoptosis-resistant cells that limits further recovery. Other studies have noted that the CD8+ T cells remain unchanged after ART and are not impacted by class of antiretrovirals used [40]. Here, we additionally note that the expanded CD8+CD28− T cell population is associated with lower CD4∶CD8 ratios, which suggests that the expansion of CD8+ T cells limits the ability other subpopulations of T cells to fully recover. Figure 6 illustrates a model how the recovery of naïve T cells and normalization of the CD4∶CD8 ratio may be impacted by several simultaneously impaired processes.

It is of interest that naïve CD8+ T cell numbers do not correlate with the CD4+ T cell nadir, length of therapy, duration of HIV infection, or total CD4+ T cell count. The lack of association between these factors and naïve CD8+ T cell recovery suggests that the damage done with uncontrolled HIV infection occurs relatively early and that recovery of T cell subpopulations, particularly naïve CD8+ T cells, may be impaired regardless of the “severity” of disease prior to treatment initiation. The impact of early ART initiation on the recovery of naïve T cell numbers remains to be seen.

One consideration for this study is the inability to closely control for other confounding factors in our HIV-uninfected control group that may influence T cell phenotypes: age, gender, co-infections, substance abuse. These analyses should be further explored in well-characterized age-matched cohorts of HIV-infected and HIV-uninfected subjects. In addition, characterization of T cell responses to neoantigens including those used in vaccinations, and/or tumor antigens may provide further insight into the impact of these immunologic parameters on development of antigen-specific T cell responses.

The inability for normalization of the CD4∶CD8 ratio in the majority of individuals on ART may reflect a persistent expansion of CD8+ T cells, particularly those of an effector phenotype and decrease in those with a naïve phenotype as we have shown. Notably, each of these immunologic characteristics has been associated with advanced aging and accelerated disease progression in elderly HIV-uninfected adults [2], [11], [37], [41]–[43]. The clinical correlation of continued immunologic perturbations is thus important to pursue given the importance of naïve T cell recovery in the defense against new pathogenic infections and tumor surveillance. It remains to be seen whether these persistent deficits portend decreased responses to vaccination and/or serve as a potential etiology for the rise in non-AIDS, age-associated clinical conditions (e.g., increased rates of cancers and of cardiovascular disease) among HIV-infected subjects. Further studies using large cohorts are needed to evaluate the clinical profile of individuals with different immune phenotypes so that these aberrant phenotypes can be validated as biomarkers of clinical risk as well as to better obtain an understanding of the drivers of low CD4∶CD8 ratios while receiving effective ART.
